# Role of long non‑coding RNA leucine‑rich repeat containing 75 A‑antisense RNA1 in the invasion and progression of renal cell carcinoma

**DOI:** 10.3892/or.2024.8844

**Published:** 2024-11-14

**Authors:** Takanori Tokunaga, Hiroshi Hirata, Yukihiro Hitaka, Nakanori Fujii, Keita Kobayashi, Takahide Hayano, Yoshiyuki Asai, Koji Shiraishi

**Affiliations:** 1 Department of Urology, Graduate School of Medicine, Yamaguchi University, Ube, Yamaguchi 755-8505, Japan; 2 Department of Systems Bioinformatics, Graduate School of Medicine, Yamaguchi University, Ube, Yamaguchi 755-8505, Japan; 3 The Division of Systems Medicine and Informatics, Research Institute for Cell Design Medical Science, Yamaguchi University, Ube, Yamaguchi 755-8505, Japan; 4 AI Systems Medicine Research and Training Center, Graduate School of Medicine, Yamaguchi University and Yamaguchi University Hospital, Ube, Yamaguchi 755-8505, Japan

**Keywords:** leucine-rich repeat containing 75 A-antisense RNA1, microRNA-370-5p, renal cancer, RCC, lncRNA

## Abstract

Long noncoding RNAs (lncRNAs) serve pivotal roles in cancer biology. The present study investigated the oncogenic roles of lncRNAs in renal cell carcinoma (RCC) and their potential as prognostic biomarkers. The lncRNA leucine-rich repeat containing 75 A-antisense RNA1 (LRRC75A-AS1) was identified through lncRNA microarray as a potential lncRNA that may predict the efficacy of immune checkpoint inhibitor therapy and cancer progression in RCC. The present study subsequently assessed the expression of LRRC75A-AS1 in 212 patients with clear cell RCC (ccRCC) who underwent nephrectomy, and performed *in vitro* functional analysis of LRRC75A-AS1 in RCC cell lines. Additionally, the interactions between LRRC75A-AS1, microRNA (miR)-370-5p and ADAMTS5 were explored. LRRC75A-AS1 was revealed to be significantly upregulated in ccRCC tissues compared with in adjacent normal tissues, and high LRRC75A-AS1 expression was associated with poor prognosis, including lower progression-free survival, in patients with RCC. The knockdown of LRRC75A-AS1 in RCC cell lines resulted in reduced cell proliferation and invasion, highlighting its role in promoting tumorigenesis. Furthermore, the interaction among LRRC75A-AS1, miR-370-5p and ADAMTS5 was suggested as a regulatory mechanism underlying RCC progression. These findings indicated that LRRC75A-AS1 may function as an oncogene in RCC, promoting cell proliferation and invasion. Its significant upregulation in ccRCC tissues and association with poor prognosis underscore its potential as a prognostic biomarker for RCC. Understanding the regulatory interactions among LRRC75A-AS1, miR-370-5p and ADAMTS5 may provide new insights into the molecular mechanisms underlying RCC and facilitate the identification of novel therapeutic targets.

## Introduction

Renal cell carcinoma (RCC) is a renal malignancy originating from the epithelium of the renal tubules of nephrons, which accounts for 2–3% of adult malignancies and 80–90% of adult renal malignancies globally (1,2). RCC is often asymptomatic until its advanced stages, with ~30% of patients diagnosed at the metastatic stage (3). Despite being the third most common urogenital malignancy after prostate and bladder cancer, RCC has a high mortality rate (4), and its incidence and mortality rates are increasing worldwide annually.

Clear cell RCC (ccRCC) is the predominant histological subtype of RCC, accounting for ~75% of all RCC cases (2). The current treatment for localized RCC is partial or radical nephrectomy. However, advanced RCC is resistant to chemotherapy and radiotherapy, making treatment challenging (5,6). Recently, molecular targeted drugs and immune checkpoint inhibitors (ICIs) have emerged as significant therapeutic options in urological oncology. Randomized controlled trials have demonstrated the efficacy and safety of combination immunotherapy, particularly with ICIs and molecular targeted drugs (tyrosine kinase and multikinase inhibitors), as a first-line treatment for advanced RCC (7). Although some patients with RCC (5–15%) achieve complete remission, 30–60% do not respond to treatment, and no predictive markers currently exist to determine which patients will respond. Consequently, a number of patients experience disease progression and eventually succumb to the cancer.

Genomic studies have revealed that >90% of human genes are actively transcribed, but only 2% encode proteins. This highlights the prevalence of non-coding RNAs (ncRNAs) in the genome (8,9). ncRNAs are categorized based on size into small ncRNAs and long ncRNAs (lncRNAs), with lncRNAs being >200 nucleotides in length. Although small ncRNAs, such as microRNAs (miRNAs/miRs), have been extensively studied, and their roles in gene regulation and cellular processes in various cancer types are well-documented (9), lncRNAs have more recently garnered attention for their significant roles in normal development and disease, including cancer (10). lncRNAs are involved in various biological processes, such as epigenetic regulation, nuclear import, cell cycle control, nuclear and cytoplasmic trafficking, imprinting, cell differentiation, alternative splicing, RNA decay, and transcription and translation (11,12). Consequently, lncRNAs have emerged as serving a critical role in cancer research, with studies indicating that certain lncRNAs can function as oncogenes, tumor suppressors or both, depending on the context (13–15).

lncRNAs are crucial for regulating immune cell activation and the tumor microenvironment, potentially enhancing the efficacy of immunotherapy by modulating the expression and function of immune checkpoint molecules (16,17). For example, AGAP2-AS1 can polarize M0 macrophages to M2, promoting RCC cell proliferation, invasion and migration (18). Additionally, some lncRNAs regulate immune checkpoint expression in tumor immunity and can predict response to ICIs (19).

According to the competing endogenous RNA (ceRNA) hypothesis, lncRNAs bind to miRNAs and compete with mRNAs that share similar miRNA response elements, thereby influencing the expression of miRNA target genes. Various ceRNAs have been identified, demonstrating their ability to sequester miRNAs or block miRNA-mRNA interactions, ultimately leading to the enhanced stability and expression of mRNAs (17).

Several lncRNAs (GAS5, MEG3/GTL2, HIF-1α-AS1, H19, KCQN1OT1, MALAT1 and HOTAIR) have been implicated in renal cancer (20–27). However, numerous lncRNAs remain uncharacterized. In the present study, the lncRNA expression in RNA extracted from the surgical specimens of patients who responded and did not respond to ICI therapy was initially analyzed. The lncRNA leucine-rich repeat containing 75 A-antisense RNA1 (LRRC75A-AS1) has been proposed as a potential prognostic marker in RCC treatment based on its expression patterns in patients with differing responses to ICI therapy.

It has previously been reported that LRRC75A-AS1 is located on chromosome 17 and predominantly acts in the cytoplasm (28). Subcellular fractionation data have indicated that cytoplasmic lncRNAs primarily function by suppressing miRNAs. Notably, previous studies have only examined the role of LRRC75A-AS1 in colorectal cancer, cervical cancer, triple-negative breast cancer and neuroblastoma (29–32); however, its mechanisms and association with ICI responses remain unclear, including in RCC.

The present study aimed to investigate whether the high expression of LRRC75A-AS1 in RCC is associated with responsiveness to ICI therapy or RCC progression, thereby affecting prognosis. The role of LRRC75A-AS1 as a novel biomarker was examined and functional analyses were conducted using immortalized RCC cell lines. To the best of our knowledge, the present study is the first to suggest that LRRC75A-AS1 may serve as a novel biomarker for RCC, influencing cancer invasion and metastasis.

## Materials and methods

### Clinical samples

The present study included 212 patients who underwent partial or radical nephrectomy at Yamaguchi University Hospital (Ube, Japan) between October 2005 and December 2022. Tumor tissue samples were collected from all patients, while adjacent normal tissue samples were collected from a subset of patients (n=25) for further analysis. All patients were pathologically diagnosed with ccRCC. Blood samples were collected preoperatively and 7 days postoperatively for the analysis of clinical data, such as neutrophil-to-lymphocyte ratio (NLR). NLR was calculated by dividing the absolute neutrophil count by the absolute lymphocyte count. Among these patients, 10 underwent ICI therapy, with renal cancer tissue samples from five effective and five ineffective patients available for analysis. The detailed patient characteristics are presented in Table I.

### lncRNA expression profiling using microarray

To identify lncRNAs that may predict the efficacy of ICI therapy in patients with ccRCC, a Human V5.0 LncRNA Array Service (cat. no. AS-S-LNC-H; ArrayStar Inc.) was performed on formalin-fixed paraffin-embedded (FFPE) samples (4 µm; fixed in 10% neutral-buffered formalin at room temperature for 48 h) from patients who continued ICI therapy for 2 years (n=2) and those who experienced disease progression within 6 months after ICI therapy (n=2). After nephrectomy, samples of renal cancer tissues were collected from all 212 patients, while adjacent normal renal tissues were obtained from a subset of 25 patients. One part was stored at −80°C in RNAlater to stabilize and protect RNA through immediate RNase inactivation, while the other part was processed into FFPE blocks. The FFPE sections were stained with hematoxylin and eosin using the Tissue-Tek Prisma Plus system (Sakura Finetek Japan). Hematoxylin staining was performed at room temperature for 8 min, followed by eosin staining for 6 min, according to the standard protocol. The sections were then microdissected to identify cancerous and normal tissues. An optical microscope (Nikon Corporation) was used for the observation. RNA was extracted from each of these microdissected tissues using the Maxwell^®^ RSC simplyRNA Tissue Kit (cat. no. AS1340; Promega Corporation). The total RNA concentration was measured using NanoDrop One (Thermo Fisher Scientific, Inc.). Total RNA purity was confirmed by measuring the RNA integrity number equivalent. Using 100 ng total RNA, the samples were processed for hybridization onto the Arraystar Human LncRNA Expression Array V5.0 platform. This platform covers 39,317 well-annotated lncRNAs. The extracted RNA was labeled with Cy3 using the Arraystar RNA Labeling Kit (Arraystar Inc), and then hybridized onto the lncRNA microarray slides according to the manufacturer's protocol. After hybridization, the slides were washed and scanned using the Agilent G2565CA Microarray Scanner System (Agilent Technologies, Inc.). The raw signal intensities were extracted using Agilent Feature Extraction software (v11.0.1.1; Agilent Technologies, Inc.) and data analysis was performed using GeneSpring GX software (v12.1; Agilent Technologies, Inc.). The raw data were normalized using the quantile normalization method, and differentially expressed lncRNAs were identified by comparing the two patient groups. Statistical significance was determined using an unpaired Student's t-test, and lncRNAs with a fold change >1.8 and a P<0.5 were considered significantly differentially expressed.

### Cell culture

Renal cancer cell lines [769-P (ATCC no. CRL-1,933), 786-O (ATCC no. CRL-1,932), ACHN (ATCC no. CRL-1,611) and A498 (ATCC no. HTB-44)] were purchased from American Type Culture Collection. The 769-P, 786-O, ACHN and A498 cell lines were cultured in Roswell Park Memorial Institute 1640 medium (Gibco; Thermo Fisher Scientific, Inc.), supplemented with 10% fetal bovine serum (cat. no. 172012; Nichirei Biosciences Inc.), and penicillin G (100 U/ml) and streptomycin sulfate (0.1 mg/ml) (cat. no. A5955; Sigma-Aldrich; Merck KGaA), and were maintained in a humidified incubator at 37°C with 5% CO_2_.

### Total RNA extraction from tissues and cells

Total RNA was extracted from human renal cancer tissues and adjacent non-cancerous normal renal tissues using the miRNeasy FFPE Kit (Qiagen GmbH) after pathological examination and the FFPE method. Additionally, RNA (miRNA and total RNA) was extracted from frozen tissues and RCC cell lines using the PureLink^®^ RNA Mini Kit (Invitrogen; Thermo Fisher Scientific Inc.) according to the manufacturer's protocol.

### cDNA synthesis

cDNA was synthesized from the extracted RNA by reverse transcription (RT) using the PrimeScript RT Reagent Kit (Takara Biotechnology Co., Ltd.), following the manufacturer's instructions, and was subsequently used for quantitative (q)PCR.

### Knockdown of LRRC75A-AS1 in RCC cells

The 769-P and 786-O cells were transfected with LRRC75A-AS1 small interfering (si)RNA (si-LRRC75A-AS1; cat. no. n547418) or a negative control siRNA [si-negative control (si-NC); cat. no. 4390843] (both from Thermo Fisher Scientific, Inc.) following the manufacturer's instructions. Briefly, the cells were grown in six-well plates at a density of 0.25–1×10^6^ cells/well and transfected individually with si-LRRC75A-AS1 at a concentration of 50 pmol/well. The transfection was performed at 37°C and incubated for 72 h. The effect of si-LRRC75A-AS1 knockdown was examined by reverse transcription (RT)-qPCR using RNA extracted 48 h after transfection. Cell viability was assessed 24, 48, and 72 h after transfection, and invasion 48 h after transfection. Transfection was performed using Lipofectamine™ RNAiMAX Transfection Reagent (Invitrogen; Thermo Fisher Scientific, Inc.) according to the manufacturer's instructions.

### Cell viability and cell invasion assays

Cell viability was assessed using the MTS assay (CellTiter 96 AQueous One Solution Cell Proliferation Assay; Promega Corporation) according to the manufacturer's protocol. Measurements were taken at 24, 48 and 72 h after plating by determining the optical density (OD) at 490 nm. The OD measurements were conducted in triplicate.

Cell invasion was assessed using the CytoSelect 24-well Cell Invasion Assay Kit (Cell Biolabs Inc.) on a 24-well Transwell plate (pore size, 8 µm). The chambers were coated with Matrigel at room temperature for 1 h. The transfected cells were transferred to the upper chamber in triplicate at a cell density of 0.5–1.0×10^6^ cells/ml; a total of 300 µl cell suspension was added to each well, resulting in 1.5–3.0×10^5^ cells/well. After 48 h of incubation at 37°C (5% CO_2_), the cells that migrated through the membrane were stained. Extraction was performed using the Extraction Solution from the kit, and the results were expressed as the number of invaded cells. Quantification was performed by measuring the OD at 560 nm using a plate reader, according to the manufacturer's instructions.

### qPCR

qPCR was performed in triplicate using the Applied Biosystems StepOnePlus and TaqMan Universal PCR Master Mix (both from Applied Biosystems; Thermo Fisher Scientific, Inc.), according to the manufacturer's protocols. TaqMan probes and primers were also purchased from Applied Biosystems; Thermo Fisher Scientific, Inc. Human β-actin (Assay ID: Hs01060665_g1) and human RNU48 (Assay ID: 001006) were used as endogenous controls. RNU48 was used as a control for miRNA, while human β-actin was used as a control for LRRC75A-AS1 and ADAMTS5. The expression levels of lncRNA LRRC75A-AS1 (Assay ID: Hs00415106_m1), hsa-miR-370-5p (Assay ID: 462392_mat) and ADAMTS5 (Assay ID: Hs01095518_m1) were determined using StepOnePlus software (version 2.1; Applied Biosystems; Thermo Fisher Scientific, Inc.) and the 2^−ΔΔCq^ method (33). The following thermocycling conditions were used: For lncRNA and ADAMTS5, initial denaturation at 95°C for 20 sec, followed by 40 cycles at 95°C for 1 sec and 60°C for 20 sec; for miRNA, initial denaturation at 95°C for 20 sec, followed by 40 cycles at 95°C for 3 sec and 60°C for 30 sec.

### Bioinformatics analysis

To identify miRNAs with strong binding affinity to LRRC75A-AS1, the miRNA target scanner miRanda (http://www.microrna.org/) was used (34). The Ensembl Canonical cDNA sequence (https://rapid.ensembl.org/index.html) for LRRC75A-AS1 (ENST05220043563.1; 3,310 bp) and mature miRNA sequences for *Homo sapiens* from miRBase (35) were used as input for miRanda. The mature miRNA sequences for *Homo sapiens* used as input of miRanda were extracted from the mature miRNA sequences file downloaded from the miRBase (https://www.mirbase.org/download/). Pairing score ≥160 and energy score ≤-20 were used as cutoff thresholds for miRanda analysis. Target genes for miRNAs were searched through TargetScanHuman (https://www.targetscan.org/vert_80/) and miRDB (https://mirdb.org/mirdb/index.html).

### Statistical analysis

Continuous variables were compared using the unpaired Student's t-test, the paired Student's t-test for matched samples, or the Mann-Whitney U test. Survival analysis was performed using the Kaplan-Meier method, and comparisons were made using the log-rank test. A Cox proportional hazards regression model was employed in both univariate and multivariate analyses to identify the risk factors for recurrence and progression. Statistical significance for functional *in vitro* analysis was determined using an unpaired Student's t-test for comparing two groups, or one-way analysis of variance (ANOVA) followed by the Tukey post hoc test for more than two groups. Statistical analyses were performed using JMP software (Pro.16; SAS Institute, Inc.). All numerical data are presented as the mean ± standard deviation. P-values were two-sided, and P<0.05 was considered to indicate a statistically significant difference.

## Results

### lncRNA microarray data analysis of RCC

**Figure 1.**
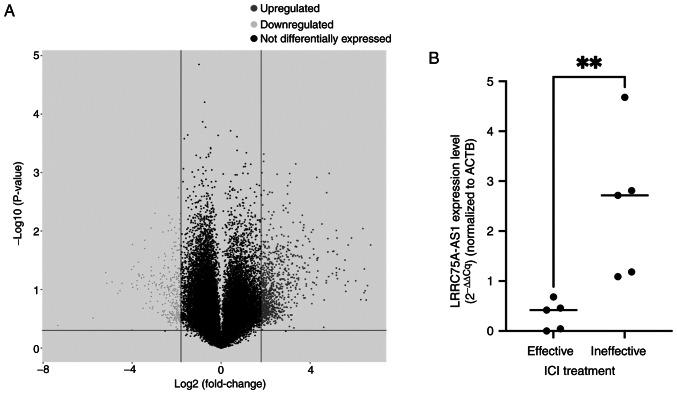
Differential expression of lncRNAs and the expression of LRRC75A-AS1 in patients with ccRCC. (A) Volcano plot of differentially expressed lncRNAs comparing patients with ccRCC who received ICI therapy for 2 years (n=2) and those who developed progressive disease within 6 months after ICI therapy (n=2). lncRNA microarray identified several lncRNAs highly expressed in ICI-ineffective patients. (B) Reverse transcription-quantitative PCR analysis of LRRC75A-AS1 expression in ccRCC tissues. The expression of LRRC75A-AS1 was significantly higher in ccRCC tissues from the ICI ineffective group (n=5) than in the effective group (n=5). **P<0.001. ccRCC, clear cell renal cell carcinoma; lncRNA, long non-coding RNA; LRRC75A-AS1, leucine-rich repeat containing 75 A-antisense RNA1; ICI, immune checkpoint inhibitor.

The lncRNA microarray analysis identified several lncRNAs that were highly expressed in patients who were unresponsive to ICI therapy (Fig. 1A). However, the present study focused on lncRNA LRRC75A-AS1 due to the limited existing literature on this particular lncRNA.

### LRRC75A-AS1 expression in ccRCC tissues between ICI effective and ineffective groups

RT-qPCR was performed to determine whether LRRC75A-AS1 expression was upregulated in human ccRCC tissues. Although the sample size was small, the expression of LRRC75A-AS1 was significantly higher in ccRCC tissues from the ICI ineffective group (n=5) compared with that in the effective group (n=5) (P<0.01; Fig. 1B).

### Relationship between LRRC75A-AS1 expression levels and the prognosis of patients with RCC

**Figure 2.**
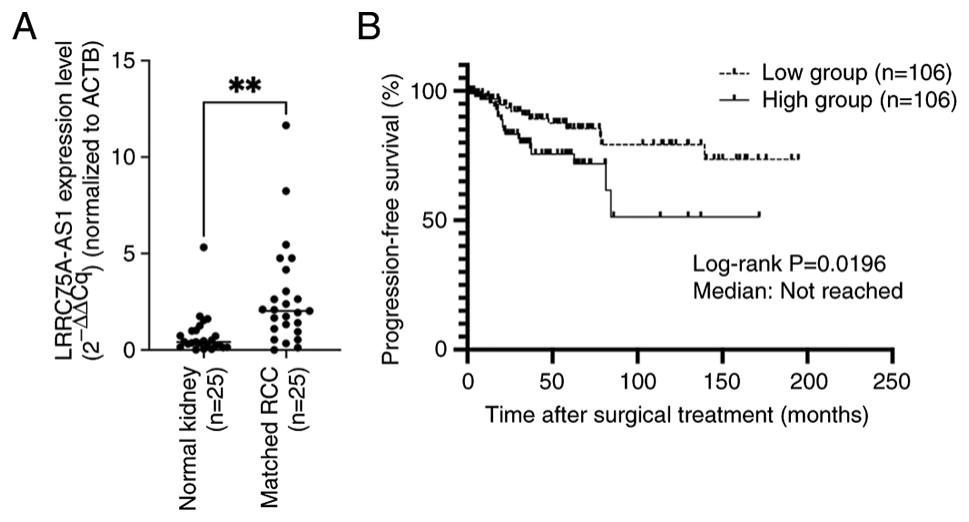
Association of LRRC75A-AS1 expression with clinical outcomes in renal cancer. (A) Quantitative comparison of LRRC75A-AS1 expression levels between matched normal renal tissues and RCC tissues using reverse transcription-quantitative PCR. Notably, LRRC75A-AS1 expression was significantly elevated in RCC tissues than in their normal counterparts. **P<0.01. (B) Kaplan-Meier plot showing the PFS of patients divided into two groups (n=106/group) based on median LRRC75A-AS1 expression. The high LRRC75A-AS1 expression group had significantly worse PFS compared with the low LRRC75A-AS1 expression group (log-rank P=0.0196). LRRC75A-AS1, leucine-rich repeat containing 75 A-antisense RNA1; PFS, progression-free survival; RCC, renal cell carcinoma.

The present study compared LRRC75A-AS1 expression in 25 matched normal renal tissues and renal cancer tissues (all ccRCC) by RT-qPCR. LRRC75A-AS1 expression was significantly higher in all renal cancer tissues compared with that in the matched normal renal tissues (P=0.0016; Fig. 2A). Subsequently, the expression levels of LRRC75A-AS1 were assessed in 212 ccRCC samples, and the samples were divided into two groups based on the median expression of LRRC75A-AS1. The high LRRC75A-AS1 group exhibited significantly shorter progression-free survival (PFS) compared with the low LRRC75A-AS1 group (log-rank P=0.0196) (Fig. 2B). However, no significant difference was found in the overall survival rate between the groups (P=0.08; data not shown). Subsequently, the present study analyzed the association between LRRC75A-AS1 expression and various clinicopathological parameters (Table II). In addition, prognostic factors for PFS, including sex, age, body mass index, neutrophil-to-lymphocyte ratio, stage, Fuhrman grade, sarcomatoid features and LRRC75A-AS1 expression levels were assessed in patients with ccRCC (Table II). Elevated LRRC75A-AS1 expression emerged as a significant independent risk factor for PFS in the multivariate analysis, similar to other clinical prognostic factors (hazard ratio=2.88; P=0.006).

### Effect of LRRC75A-AS1 knockdown on cell viability and invasion in vitro

**Figure 3.**
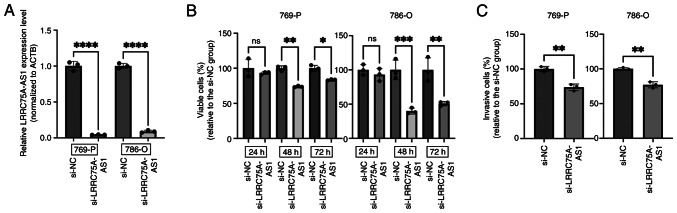
Effect of LRRC75A-AS1 knockdown on 769-P and 786-O renal cancer cell function. Two renal cancer cell lines (769-P and 786-O) were transiently transfected with either si-LRRC75A-AS1 or si-NC. (A) Validation of LRRC75A-AS1 knockdown in RCC cell lines. LRRC75A-AS1 expression was significantly decreased after siRNA transfection. (B) Cell viability was assessed using an MTS assay. Downregulation of LRRC75A-AS1 significantly suppressed cell proliferation in both cell lines 48 h post-transfection. (C) Invasion assay. Knockdown of LRRC75A-AS1 significantly reduced the invasive capacity of both cell lines compared with si-NC. *P<0.05, **P<0.01, ***P<0.001, ****P<0.0001. LRRC75A-AS1, leucine-rich repeat containing 75 A-antisense RNA1; NC, negative control; si, small interfering.

The present study analyzed and compared the expression levels of LRRC75A-AS1 in four RCC lines (769-P, 786-O, ACHN and A498) using RT-qPCR. Among these, 769-P and 786-O cells exhibited the highest expression of LRRC75A-AS1 and were selected for further experiments (Fig. S1). In 769-P and 786-O cells, LRRC75A-AS1 expression was significantly decreased after si-LRRC75A-AS1 transfection (P<0.0001; Fig. 3A). The MTS assay results revealed that the knockdown of LRRC75A-AS1 significantly suppressed cell proliferation in both cell lines 48 h post-transfection, compared with that in the si-NC group (P<0.01 and P<0.001, respectively; Fig. 3B). In the invasion assay, LRRC75A-AS1 knockdown significantly reduced the invasive capacity of 769-P and 786-O cells compared with that in the si-NC group (P<0.01; Fig. 3C). These results indicated that the knockdown of LRRC75A-AS1 expression significantly inhibited the proliferation and invasion of RCC cell lines *in vitro*.

### Interaction among LRRC75A-AS1, miR-370-5p and ADAMTS5 in RCC

**Figure 4.**
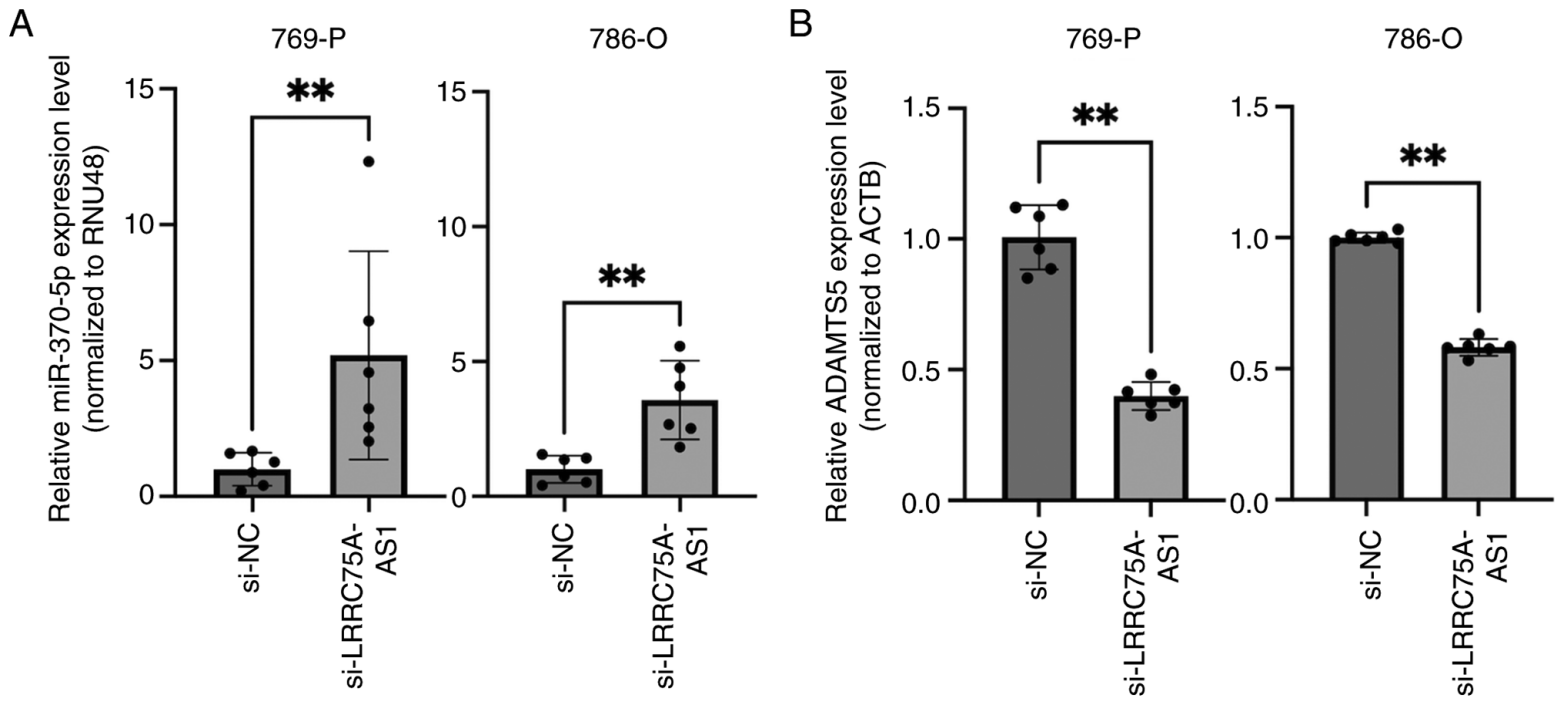
Effect of LRRC75A-AS1 knockdown on miR-370-5p and ADAMTS5 expression in the 769-P and 786-O RCC cell lines. (A) Knockdown of LRRC75A-AS1 significantly increased miR-370-5p expression in both cell lines. (B) Knockdown of LRRC75A-AS1 significantly decreased ADAMTS5 expression in both cell lines. **P<0.01. LRRC75A-AS1, leucine-rich repeat containing 75 A-antisense RNA1; miR, microRNA; NC, negative control; si, small interfering.

The present study investigated the potential relationship between LRRC75A-AS1 and miRNAs. Cytoplasmic lncRNAs act as miRNA sponges; therefore, it was hypothesized that LRRC75A-AS1 may function as a miRNA regulator. A total of 66 miRNAs identified by bioinformatics analysis as having potential binding sites on LRRC75A-AS1 were selected (Table SI). Among these, miR-370-5p functions as a tumor suppressor in renal cancer (36). Therefore, the present study focused on miR-370-5p and investigated its interaction with LRRC75A-AS1 in a renal cancer cell line. LRRC75A-AS1 knockdown significantly increased the expression of miR-370-5p in the 769-P and 786-O cell lines (P<0.01; Fig. 4A). These results suggested a reciprocal interaction between LRRC75A-AS1 and miR-370-5p. To identify the potential target genes of miR-370-5p, public databases such as miRDB and TargetScanHuman were utilized. Based on its high TargetScores and relevance to cancer, particularly as an unfavorable prognostic marker of RCC, ADAMTS5 was selected for further investigation. The expression levels of ADAMTS5 were compared between si-NC- and si-LRRC75A-AS1-transfected 769-P and 786-O cell lines. The results demonstrated a significant decrease in ADAMTS5 expression in the si-LRRC75A-AS1 group (P<0.01; Fig. 4B). These findings suggested that LRRC75A-AS1 may regulate ADAMTS5 expression through a sponge effect on miR-370-5p, thereby contributing to RCC progression. Specifically, LRRC75A-AS1 acted as a molecular sponge for miR-370-5p, preventing miR-370-5p from binding to ADAMTS5 mRNA. This inhibition could lead to increased ADAMTS5 expression, which is implicated in RCC progression and metastasis. The present results demonstrated that LRRC75A-AS1, through its sponging effect on miR-370-5p, may serve a critical role in the regulation of these target genes, contributing to the pathogenesis of RCC.

## Discussion

The present study investigated the oncogenic role of lncRNA LRRC75A-AS1 in RCC and its potential as a prognostic biomarker. Based on the lncRNA microarray data, the study focused on LRRC75A-AS1, which, to the best of our knowledge, had not previously been investigated in RCC. Using tissue samples obtained post-surgery, it was demonstrated that high LRRC75A-AS1 expression in RCC was associated with a poor prognosis. Specifically, high LRRC75A-AS1 expression was significantly associated with PFS and served as an independent predictor, similar to clinical prognostic factors such as Fuhrman grade, stage (37,38), and sarcomatoid differentiation. Additionally, the expression levels of LRRC75A-AS1 were examined in four RCC cell lines. Using specific siRNAs to knock down LRRC75A-AS1 expression in 769-P and 786-O cell lines, a reduction in cell proliferation and invasion was observed. These results suggested that LRRC75A-AS1 may function as an oncogene, contributing to RCC development.

Despite their limited protein-coding abilities, lncRNAs regulate the stemness of cancer stem cells (CSCs) and mediate chemoresistance (39). A previous review discussed the critical roles of various lncRNAs in maintaining CSC properties and mediating resistance to chemotherapy (40). Notable lncRNAs, such as HOTAIR, MALAT1 and H19, were highlighted for their involvement in these processes. These lncRNAs contribute to CSC maintenance and drug resistance by interacting with key signaling pathways, such as Wnt/β-catenin, Notch and Hedgehog. In RCC, lncRNAs have also been implicated in sunitinib resistance. For example, lncRNA IGFL2-AS1 can induce sunitinib resistance through extracellular vesicles (41). Furthermore, LRRC75A-AS1, the central focus of the present study, may be associated with these processes, particularly CSC maintenance and chemoresistance, through pathways such as Wnt/β-catenin, Notch and Hedgehog. Continued research in this area is essential to address the challenges associated with RCC treatment.

The present study also examined the potential interactions between LRRC75A-AS1 and other ncRNAs, such as miRNAs. lncRNAs can influence post-transcriptional regulation by competing for shared miRNA response elements and acting as natural miRNA sponges, thereby reducing the binding of endogenous miRNAs to their target genes (42). Cytoplasmic lncRNAs and miRNAs have been implicated in post-transcriptional regulation across various types of cancer, including RCC, through their sponge effects. Although the mechanisms by which LRRC75A-AS1 operates in RCC remain unexplored, LRRC75A-AS1 has been identified as a ceRNA in triple-negative breast cancer. Specifically, LRRC75A-AS1 has been shown to regulate miR-30a-5p through a sponge effect, resulting in increased BAALC expression, and contributing to cancer proliferation and invasion (31). The present study identified the miRNAs that may bind to LRRC75A-AS1. Among these, miR-370-5p emerged as a significant miRNA in RCC and is located in the cytoplasm of renal cancer cells (36). Various studies have shown that miR-370-5p may act as a tumor suppressor in various types of cancer, including RCC, and lung (43), colorectal (44) and nasopharyngeal (45) cancer. For example, low miR-370-5p expression levels have been observed in RCC cells; by contrast, the artificial upregulation of miR-370-5p expression has been shown to mitigate the pro-tumor effects of circCOL5A1 by suppressing tumor malignancy and glycolysis (36).

In the present study, a significant association was observed between miR-370-5p and LRRC75A-AS1 expression, suggesting a potential regulatory interaction. This interaction supports the ceRNA hypothesis indicating that LRRC75A-AS1 acts as a sponge for miR-370-5p, thereby regulating its target mRNAs involved in RCC progression. Specifically, the present cellular experiments revealed that the siRNA-mediated knockdown of LRRC75A-AS1 led to increased miR-370-5p expression, consistent with its role as a tumor suppressor. These results suggested that LRRC75A-AS1 may suppress miR-370-5p expression, thereby contributing to RCC progression. Understanding these interactions could provide new insights into the molecular mechanisms driving RCC and facilitate the identification of novel therapeutic targets. Using the miRDB and TargetScanHuman databases, the target genes of miR-370-5p were predicted and ADAMTS5 was identified as a candidate mRNA. ADAMTS5 is involved in extracellular matrix remodeling, and promotes tumor invasion and metastasis in various cancer types (46). Notably, high ADAMTS5 expression in hepatocellular carcinoma has been associated with a poor prognosis (47). These findings suggested that ADAMTS5 may be regulated by miR-370-5p in RCC, contributing to tumor malignancy. The sponging effect of LRRC75A-AS1 on miR-370-5p could result in increased ADAMTS5 expression, thereby facilitating RCC progression. Understanding this interaction could provide new insights into the molecular mechanisms driving RCC and present innovative therapeutic targets.

A limitation of the present study was the small number of patients treated with ICIs, which limits the generalizability of the findings. Further studies are warranted to fully elucidate the role of LRRC75A-AS1 in ICI therapy. The limited sample size hinders the ability to draw definitive conclusions regarding the interaction between LRRC75A-AS1 expression and ICI treatment response. Future studies with a larger cohort of patients treated with ICIs are essential to validate these findings and to explore the potential of LRRC75A-AS1 as a predictive biomarker for ICI therapy response.

Another limitation is the lack of *in vivo* experiments, such as those utilizing an orthotopic animal model with siRNA- or short hairpin RNA-induced LRRC75A-AS1 knockdown. Such studies would aid in elucidating the molecular mechanisms involved in RCC invasion and metastasis. Additionally, siRNA was used in cell lines to investigate the effect of LRRC75A-AS1 on cell proliferation and invasion *in vitro*, obtaining significant results; however, overexpression experiments have not yet been conducted. Furthermore, the direct interaction between LRRC75A-AS1 and miR-370-5p has not been experimentally validated, which is another limitation of the present study. Similarly, the direct interaction between miR-370-5p and ADAMTS5 has not been experimentally validated, such as via a luciferase reporter assay. Future studies should include *in vitro* and *in vivo* experiments to confirm the regulatory interactions and functional effects of LRRC75A-AS1 and its target genes.

In conclusion, LRRC75A-AS1 may function as an oncogene in RCC, promoting tumorigenesis and progression. The significant upregulation of LRRC75A-AS1 in ccRCC tissues and its association with poor prognosis suggests its potential as a biomarker for RCC. Furthermore, the regulatory interaction between LRRC75A-AS1 and miR-370-5p, which may influence the expression of ADAMTS5, underscores their roles in RCC progression. Specifically, the increased expression of ADAMTS5 facilitated by the LRRC75A-AS1-mediated sponging of miR-370-5p highlights a potential pathway contributing to tumor invasion and metastasis. Understanding this interaction and its impact on ADAMTS5 expression could provide new insights into the molecular mechanisms underlying RCC development and identify novel therapeutic targets. Continued research into the molecular mechanisms of LRRC75A-AS1, including its effect on ADAMTS5 and its clinical applications, could significantly improve RCC management and patient outcomes.

## Supplementary Material

Supporting Data

Supporting Data

## Figures and Tables

**Table I.** Characteristics of 212 patients who underwent partial or radical nephrectomy.

Variable	Value
Sex, n (%)	
Male	139 (65.6)
Female	73 (34.4)
Age, years	
Median	67
Range	28-92
Pathological T stage, n (%)	
1a	124 (58.5)
1b	31 (14.6)
2a	10 (4.7)
3a	41 (19.3)
3b	5 (2.4)
4	1 (0.5)
Fuhrman grade	
G 1/2	184 (86.8)
G 3/4	28 (13.2)
Follow-up duration, months	
Median	37.1
Range	0.6–194.6
Progression	
No	178 (84.0)
Yes	34 (16.0)
Overall survival	
No	193 (91.0)
Yes	19 (9.0)

**Table II.** Univariate and multivariate analyses of predictive factors of progression-free survival.

	Univariate			Multivariate		
		
Parameter	HR	95% CI	P-value	HR	95% CI	P-value
Sex, male vs. female	0.69	0.74–2.85	0.278			
Age, ≥65 vs. <65 years	0.95	0.48–1.88	0.888			
BMI, ≥25 vs. <25 kg/m^2^	0.79	0.39–1.63	0.526			
NLR, ≥3 vs. <3	1.03	0.51–2.10	0.888			
Stage, ≥II vs. I	6.49	3.16–13.35	<0.0001^a^	3.48	1.34–8.99	0.01^a^
Metastasis, ≥1 vs. 0	2.59	0.79–8.52	0.117			
Fuhrman grade, ≥3 vs. <3	4.38	2.12–9.06	<0.0001^a^	3.2	1.37–7.46	0.007^a^
Sarcomatoid, yes vs. no	4.68	2.12–9.07	0.0003^a^	3.77	1.60–8.89	0.003^a^
LRRC75A-AS1, high vs. low	2.26	2.03–10.78	0.023^a^	2.88	1.35–6.14	0.006^a^

a P<0.05. BMI, body mass index; CI, confidence interval; HR, hazard ratio; LRRC75A-AS1, leucine-rich repeat containing 75 A-antisense RNA1; NLR, neutrophil-to-lymphocyte ratio.

## Data Availability

The microarray data generated in the present study may be found in the Gene Expression Omnibus under accession number GSE276355 or at the following URL: https://www.ncbi.nlm.nih.gov/geo/query/acc.cgi?acc=GSE276355. The other data generated in the present study may be requested from the corresponding author.

## Abbreviations

ccRCC: clear cell renal cell carcinoma
ceRNA: competing endogenous RNA
ICI: immune checkpoint inhibitor
lncRNA: long non-coding RNA
miRNA: microRNA
ncRNA: non-coding RNA
PFS: progression-free survival
RCC: renal cell carcinoma
si-NC: small interfering RNA-negative control
CSCs: cancer stem cells
FFPE: formalin-fixed paraffin-embedded
NLR: neutrophil-to-lymphocyte ratio
