# Supplementary material for: Role of long non‑coding RNA leucine‑rich repeat containing 75 A‑antisense RNA1 in the invasion and progression of renal cell carcinoma
Source: Oncol Rep. 2024 Nov 14;53(1):11. doi: 10.3892/or.2024.8844 (PMC11603548; doi:10.3892/or.2024.8844)

Figure S1. Differential expression of LRRC75A-AS1 in renal cancer cell lines. Comparison of LRRC75A-AS1 expression levels among various renal cancer cell lines (769-P, 786-O, ACHN and A498). LRRC75A-AS1 expression was significantly higher in 769-P and 786-O cells than in other renal cancer cell lines. ***P<0.001, ****P<0.0001. LRRC75A-AS1, leucine-rich repeat containing 75 A-antisense RNA1.

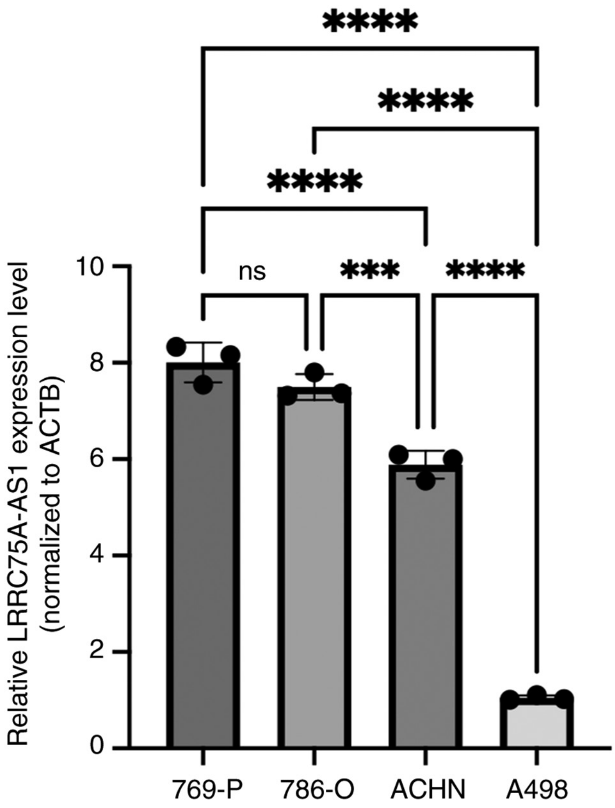

Supplement: Supporting Data [file Supplementary_Data1.pdf]
